# Synthesis of WS_1.76_Te_0.24_ alloy through chemical vapor transport and its high-performance saturable absorption

**DOI:** 10.1038/s41598-019-55755-x

**Published:** 2019-12-19

**Authors:** Zhengting Du, Chi Zhang, Mudong Wang, Xuejin Zhang, Jian Ning, Xinjie Lv, Gang Zhao, Zhenda Xie, Jinlong Xu, Shining Zhu

**Affiliations:** 1 College of Engineering and Applied Sciences, Nanjing University, Nanjing, 210093 China; 2 School of Physics, Nanjing University, Nanjing, 210093 China; 3 School of Electronic Science and Engineering, Nanjing University, Nanjing, 210093 China

**Keywords:** Optical materials and structures, Nanoscale materials

## Abstract

Layered transitional metal dichalcogenides (TMDs) are drawing significant attentions for the applications of optics and optoelectronics. To achieve optimal performances of functional devices, precisely controlled doping engineering of 2D TMDs alloys has provided a reasonable approach to tailor their physical and chemical properties. By the chemical vapor transport (CVT) method and liquid phase exfoliation technique, in this work, we synthesized WS_1.76_Te_0.24_ saturable absorber (SA) which exhibited high-performance of nonlinear optics. The nonlinear saturable absorption of the WS_1.76_Te_0.24_ SA was also measured by the open aperture Z-scan technique. Compared to that of the binary component WS_2_ and WTe_2_, WS_1.76_Te_0.24_ SA has shown 4 times deeper modulation depth, 28% lower saturable intensity and a much faster recovery time of 3.8 ps. The passively Q-switched laser based on WS_1.76_Te_0.24_ was more efficient, with pulse duration narrowed to 18%, threshold decreased to 28% and output power enlarged by 200%. The promising findings can provide a method to optimize performances of functional devices by doping engineering.

## Introduction

2D transitional metal dichalcogenides (TMDs) materials with formula MX_2_ (M = Mo, W; X = S, Se, Te) have attracted intense attentions thanks to their unique layered structures, attractive carrier mobility, adjustable bandgap, strong light-matter interaction and stable chemical properties^1–9^. The abundant physical and chemical properties of TMDs are widely applied to photodetectors, logic devices, memories, catalysis, transistors and lasers. The discrepancy of strong covalent bonds intra layers and weak van der Waals inter layers allows down-top and top-down methods to obtain the 2D TMDs nanosheets. For example, chemical vapor deposition (CVD) and wet chemical synthesis are used to obtain the 2D TMDs nanosheets by the methods of down-top^10,11^. It is also desirable to combine two different mature methods of chemical vapor transport (CVT) and mechanical stripping to achieve the nanomaterials (top-down)^12,13^. TMDs can potentially serve as SAs, such as molybdenum disulfide and tungsten disulfide, which already exhibited promising applications with much larger modulation depth than graphene^14–18^. The band-gaps of MoS_2_ and WS_2_ are about 1.6~1.9 eV in the visible range, indicating the natural MoS_2_ and WS_2_ are far from serving as saturable absorbers in highly developed infrared lasers. In recent years, the defects have been introduced into TMDs in order to extend the bandwidth of TMDs SAs. It has been proved by theories and experiments that M or X vacancy defects in TMDs can reduce their band-gaps^19–22^. S. Wang reduced the band-gap of MoS_2_ by introducing defects^19^. MoS_2_ with S defects can be used as a broadband saturable absorber. The passively Q-switched lasers in the range of 1.06, 1.42 and 2.1 μm were realized based on the MoS_2_ SA with S defects, corresponding pulse duration of 970, 729 and 410 ns, respectively^19^. X. Guan *et al*. experimentally realized 2.7 μm self-Q-switching laser of Er:Y_2_O_3_ based on WS_2_ SA with pulse width of 1.36 µs^20^. The passively Q-switched lasers at the wavelengths of 1.5, 2.0, 2.7 μm are based on the binary TMDs with defects^14–22^. However, M or X vacancy defects in MX_2_ make its structure unstable, so M or X vacancy defects in TMDs are difficult to repeatedly synthesized. Furthermore it is less feasible to regulate intentional defects, for example, the 2D MX_2_ often appears to be both triangle and hexagon due to the local condition. Therefore, there is an urgent need to tune the band-gaps of the TMDs so that they can perform stably in the near-infrared and mid-infrared range.

Inspired by the history of Si semiconductor, the doping engineering appears to be the key to tailor physical and chemical properties of TMDs. Mixed chalcogenides or mixed metal elements of two different TMDs can control the band gap, such as WS_2×_Se_2(1−x)_^23^ and Mo_x_W_1−x_S_2_^24^. However, it has been proven that only a few hundred milli-electron volts (meV) could be realized, i.e. 300 meV for WS_2x_Se_2(1−x)_ and 170 meV for Mo_x_W_1−x_S_2_ solid solutions respectively. P. Yu *et al*. experimentally showed that 2H-WSe_2_ and Td-WTe_2_ can form stable layered WSe_2×_Te_2(1−x)_ alloys^25^, with a phase transition from 2H-to-Td (*x* = 1 − 0.6 for 2 H structure; *x* = 0.5 and 0.4 for 2 H and 1 Td structures; *x* = 0 − 0.3 for 1 Td structure) controlled by the complete composition. The electronic structures changing from semiconducting to metallic enable wide tunability of the optical and electronic properties. Extraordinary physical properties of alloys are needed for in-depth study where the alloys showed some unique advantages compared to 2D binary TMDs, making them fundamentally and technically important in applications of optics and optoelectronics. One of the impressive physical properties of mono- and few-layer alloy is that TMDs display surprisingly excellent nonlinear optical properties, Y. Wang *et al*. studied the nonlinear optics properties of alloys of Bi_2_Te_*x*_Se_*3-x*_ with lower saturable intensity, deeper modulation depth^26^.

TMDs alloying still remains challenging resulted from the lattice mismatch of their parent counterparts. Here we synthesized WS_1.76_Te_0.24_ alloy by doping Te^2−^ ions in WS_2_ (2 H) structure. The nonlinear optics properties of WS_1.76_Te_0.24_ SA were 4 times deeper modulation depth, 28% lower saturable intensity and a much faster recovery time of 3.8 ps compared to those of WS_2_ and WTe_2_. To find out whether pulsed laser’s performance can be promoted by alloying, passively Q-switched lasers were investigated based on WS_2_, WS_1.76_Te_0.24_ and WTe_2_ SAs at the wavelength of 1060 nm. We found that the passively Q-switched laser based on WS_1.76_Te_0.24_ was more efficient, with pulse duration narrowed to 18%, threshold decreased to 28% and output power enlarged by 200%. The promising findings can provide a method to optimize performances of functional devices by doping engineering.

## Methods

### Synthesis and characterization of WS_x_Te_2−x_ SAs

**Figure 1:**
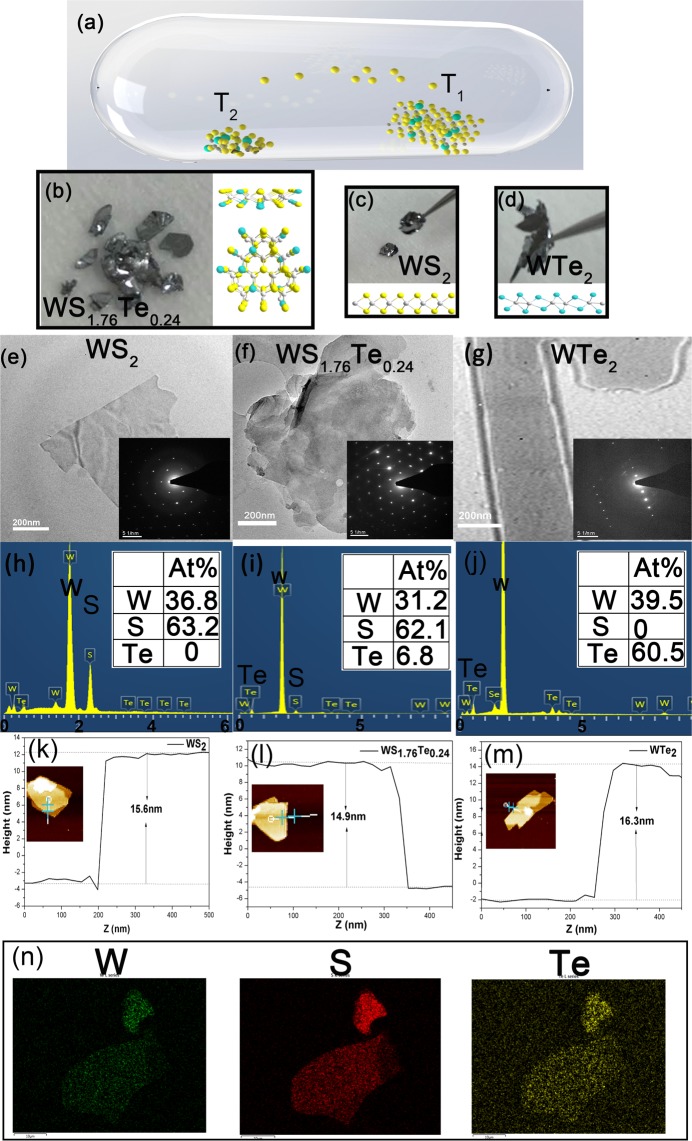
(**a**) Scheme of chemical vapor transport (CVT) for crystallization of WS_x_Te_2-x_ in a temperature gradient. Monocrystalline photograph and atomic structure of (**b**) WS_1.76_Te_0.24_. (**c**) WS_2_. (**d**) WTe_2_. TEM and SEAD characterizations of few-layer nanosheets of (**e**) WS_2_, (**f**) WS_1.76_Te_0.24_ and (**g**) WTe_2._ Corresponding full EDS scanning of (**h**) WS_2_, **(i**) WS_1.76_Te_0.24_ and (**j**) WTe_2._ AFM images and thickness measurement of typical nanosheets and height measurement recorded at different sections of (**k**) WS_2_, (**l**) WS_1.76_Te_0.24_ and (**m**) WTe_2._ (**n**) EDS mapping of WS_1.76_Te_0.24._ The clear morphology implies the uniformity of component distribution.

The WS_1.76_Te_0.24_ monocrystalline was prepared by the chemical vapor transport (CVT) method with well-controlled temperature. There were two steps to obtain WS_1.76_Te_0.24_ mono-crystalline. Firstly, WS_1.76_Te_0.24_ polycrystalline was synthesized by heating a mixture of sulphur (Strem Chemicals 99.9%), tungsten (Strem Chemicals 99.9%) and tellurium (Strem Chemicals 99.9%) with stoichiometric amounts at 750 °C for 48 hours in an evacuated and sealed quartz ampoule (8 mm ID, 10 mm OD, 300 mm length). Considering the powerful exothermicity of the reaction, the mixture was slowly preheated to 750 °C for 12 hours to avoid explosion. Secondly, WS_1.76_Te_0.24_ was grown by CVT method in a double zone furnace with as-prepared grinded polycrystalline powder and the transport agent was bromine (Sigma-Aldrich, 99.8%) at about 5 mg/mL. The procedure of growth was 72 hours in an evacuated and sealed quartz ampoule (8 mm ID, 10 mm OD, 300 mm length). Figure 1a showed a two-temperature zone tube furnace with well-controlled temperature. Throughout the growth process of WS_1.76_Te_0.24_, the raw material (T_2_) and crystal growth zones (T_1_) were kept at 1030 °C and 1010 °C, respectively. The parent components of WS_2_ and WTe_2_ were synthesized by the same method for the following contrast experiments. Figure 1b–d show the monocrystalline photographs and atomic structures of WS_1.76_Te_0.24_, WS_2_ and WTe_2_.

We prepared WS_2_, WS_1.76_Te_0.24_ and WTe_2_ SAs by liquid-phase exfoliation and spin-coating technique with the same parameters (sonication time, speed of centrifugation and spin-coating) for further comparison. First, the mixture of grinded 0.2 mg WS_1.76_Te_0.24_ monocrystalline in 4 ml acetone solvent was sonicated in high power for 40 min. Only pure acetone was employed as the solvent to avoid introduction of extra impurities. Then, we collected the one-third top of the dispersions after the centrifugation at 2500 rpm for 10 min to remove the large sedimentations. Finally, we span and coated the dispersion on SiO_2_ plate to obtain WS_1.76_Te_0.24_ SA after the acetone was easily removed by volatilization in the air.

### Material characterization

Transmission electron microscopy (TEM) and energy dispersive spectroscopy (EDS) were adopted to learn the morphology of the WS_1.76_Te_0.24_ nanoplates. TEM images in Fig. 1e–g showed the layered structures of WS_2_, WS_1.76_Te_0.24_ and WTe_2_ respectively, where the gray scale was directly proportional to the thickness. The observed well exfoliated nanoflakes with layered structure implied rigid mechanical property. The insets were the Selected Area Electron Diffraction (SAED) of WS_2_, WS_1.76_Te_0.24_ and WTe_2_ nanoflakes. The SAED showed that WS_2_, WS_1.76_Te_0.24_ and WTe_2_ were monocrystalline with 2 H, 2 H and Td phases, respectively. The diffraction of six-fold symmetry spots displayed the hexagonal lattice of WS_1.76_Te_0.24_ nanoflake. EDS measurement was also produced to determinate the element ratio of the three samples as shown in Fig. 1h–j. It can be seen that the ratio of S to Te in WS_1.76_Te_0.24_ is 1.76 to 0.24, indicating an efficient doping of Te ions in WS_2_ framework. As shown in Fig. 1k–m, the atomic force microscopy (AFM) was carried out to measure the three SAs thicknesses. The thicknesses of WS_2_, WS_1.76_Te_0.24_ and WTe_2_ nanoflakes were about 15.6, 14.9, and 16.3 nm, corresponding to 23, 19 and 27 layers, respectively. To learn more about the WS_1.76_Te_0.24_ alloy, EDS mapping was adopted shown in Fig. 1n. The green, red and yellow parts were the distribution of tungsten, sulphur, and tellurium element, respectively. The uniform doping of Te in WS_2_ of WS_1.76_Te_0.24_ was obtained. Furthermore, the WS_1.76_Te_0.24_ SA with no additional dangling bonds is stable in the air. The high chemical stability was due to the substitution of atoms in the alloy TMDs.

**Figure 2:**
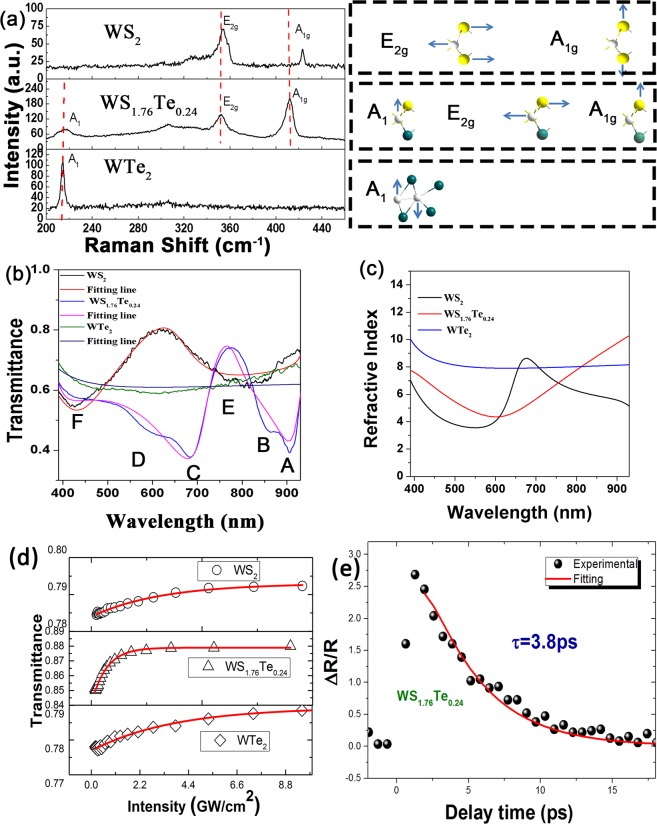
(**a**) Raman spectra and vibration modes of few-layer WS_1.76_Te_0.24_ nanosheets. (**b**) Comparison of recorded transmittance spectra and corresponding fitted line of WS_2_, WS_1.76_Te_0.24_ and WTe_2_. (**c**) The refractive index of WS_2_, WS_1.76_Te_0.24_ and WTe_2_ based on the relationship of Kramers-Kronig. (**d**)The open-aperture Z-scan measurements of WS_2,_ WS_1.76_Te_0.24_ and WTe_2_ flakes at 1060 nm. (**e**) An ultrafast signal of WS_1.76_Te_0.24_ proved that the relaxation time was ~3.8 ps.

Raman spectroscopy was employed to learn the detailed lattice vibration modes of WS_1.76_Te_0.24_ affected by doping engineering where Te^2−^ replaced the S^2−^ in the WS_2_ structure. The characterization was carried out by using a Jobin Yvon LabRam 1B Raman spectrometer with laser source at 532 nm. The comparison of the Raman fingerprints among the three samples in the range of 200–450 cm^−1^ is shown in Fig. 2a. In Fig. 2a, the characteristic peaks at 353.2 and 422.7 cm^−1^ were assigned as the in-plane (E_2g_) and out-of-plane (A_1g_) vibrational modes corresponding to WS_2_ nanoflakes. For the Td-WTe_2_, the spectrum just showed the A_1_ Raman mode at 217.8 cm^−1^. The characteristic bands of WS_1.76_Te_0.24_ showed the “two mode behavior” as the coexistence of vibrations of WS_2_ and WTe_2_. The E_2g_ and A_1g_ peaks of WS_1.76_Te_0.24_ at 352.5 and 411.5 cm^−1^ are resulted from the corresponding modes in WS_2_. The red-shift of A_1g_ (0.7 cm^−1^) and E_2g_ (11.2 cm^−1^) mode is attributed to the significant changes in the electron-phonon coupling soften by the doping of Te. The frequency broadened at A_1_ (217.8 cm^−1^) was originated from the reduced structural symmetry arising from lattice distortion due to the different atomic radius of Te and S. In earlier studies, TMDs alloys, e.g. Mo_x_W_1−x_S_2_^24^ and WS_2×_Se_2(1−x)_^23^, have the similar frequency shift. The Raman shifts further confirm the expected structural and compositional evolution in the WS_1.76_Te_0.24_ alloy.

Figure 2b showed linear transmittance spectra of WS_1.76_Te_0.24_ SA with WS_2_ and WTe_2_ as contrasts. The typical absorption peaks of WS_1.76_Te _0.24_ corresponded to the trait of TMD 2 H phase. The peaks located at the wavelength of 906 (A) and 859 nm (B) coincided with inter-band transitions. A, B are resulted from spin-orbit splitting of transitions^16^, while C and D (685 and 592 nm) are due to the higher density transition^16^. The transmittance spectrum of WS_2_ showed the TMD absorption with absorption peaks at 814 (E) and 426 (F) nm. WTe_2_ displayed semi-metallic phase absorption characteristic with broadband absorption. According to the Tauc plot by extrapolating the linear absorption versus photon energy curve, optical bandgaps of WS_1.76_Te_0.24,_ WS_2_ SAs were calculated to be 1.2 eV and 1.9 eV, respectively. The electronic structure of WS_1.76_Te_0.24_ strongly depends on the coordination environment of W and its *d*-electron counts. The Te doping in WS_2_ shorten band gap by the change coordination environment of W corresponding to the *ab initio*^23^. Thus physics properties of WS_1.76_Te_0.24_ were tailored by ion doping.

The ellipsometer is a conventional method to measure the film’s refractive index. However, ellipsometer has strict requirement on the samples for uniform surface, large size, and thin thickness. Due to its low spatial resolution, it is difficult to obtain refractive index of nanomaterials in nanometer size. Benefitting from the Lorentz-Drude model and Kramers-Kronig (K-K) relationship of the dielectric function^27^, we calculated the corresponding refractive index from the transmittance spectrum. Figure 2c shows the fitting curve of the reflectance spectra, where the refractive index parameters were obtained by the K-K relationship.

In order to understand the incorporation mechanism by Te doping into WS_2_ and the corresponding effect on optical nonlinear properties of 2D WS_1.76_Te_0.24_, we performed Z-scan measurement with a femtosecond laser (1060 nm, 175 fs) as excited source. The results of WS_2_, WS_1.76_Te_0.24_ and WTe_2_ SAs are showed in Fig. 2d. The increase of transmittance was easily observed with the increase of laser intensity, resulted from the nonlinear saturable absorption effect_._ The mechanism of saturable absorption can be explained as Pauli blocking principle in the conduction band. However, significant differences in saturable absorption efficiency and sensitivity among the three samples can be clearly distinguished in Fig. 2d. Based on the nonlinear optical theory, the transmittance is expressed in the form of^14^

1 $${\rm{T}}=1-{A}_{s}\cdot \exp (-\frac{I}{{I}_{sat}})-{A}_{ns}$$

where *A*_s_ is the modulation depth, *A*_*n*s_ is the non-saturable components, *I*_sat_ is the saturable intensity, and *I* is the incident light intensity. The data was fitted with the Eq. 1, the modulation depth and the saturable intensities were obtained as presented in Table 1. The WS_1.76_Te_0.24_ saturation intensity and modulation depth were 0.88 GW/cm^2^ and 4.47% respectively. When WS_2_ became saturated above threshold of 3.15 GW/cm^2^, the modulation depth was 1.14%. The saturation intensity and modulation depth of WTe_2_ were 3.7 GW/cm^2^ and 1.6%, respectively. Compared to binary component of WS_2_ and WTe_2_ SA, WS_1.76_Te_0.24_ SA performed 4 times deeper modulation depth and 28% lower saturable intensity, resulting from bandgap evolution of WS_1.76_Te_0.24_.

**Table 1:** SA Results for Different Two-dimensional Materials.

Materials	n	α_0_ (10^4^ cm^−1^)	α_NL_ (10^4^ cm/GW)	Imχ^(3)^ (10^−^^7^ esu)	FOM (10^−14^ cm•esu)	As (%)	I_S_ (GW/cm^2^)
WS_2_	6.19	0.90	−0.41	−4.98	5.53	1.15	3.15
WS_1.76_Te_0.24_	10.2	2.25	−0.53	−17.5	7.78	4.47	0.89
WTe_2_	8.29	1.08	−0.38	−8.28	7.66	1.60	3.70

**Figure 3:**
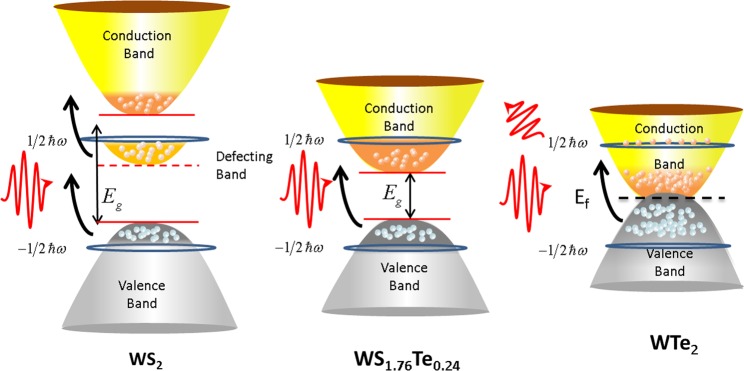
Schematic diagram of linear absorption in WS_2_, WS_1.76_Te_0.24_ and WTe_2_ SAs.

The bandgap evolution of WS_1.76_Te_0.24_ is used to interpret the lower saturable intensity. As shown in the Fig. 3, the bandgap of WS_2_ SA is 1.9 eV larger than the photon energy ($\hslash {\rm{\omega }}$) of 1060 nm laser. The saturable absorption of WS_2_ is resulted from the defect states. As shown in Fig. 3, under the excited light, the electrons in valence band of WS_2_ are transferred to the defect states. The electrons in the defect states jump to the conduction band with one more photon each. Therefore, there are two platforms that the transition of one electron from valence band to conduction band of WS_2_ and the transition requires two photons. Compared to that of WS_2_, the optical bandgap of WS_1.76_Te_0.24_ is 1.2 eV covering 1.0 µm, so the electrons directly transfer from valence band to the conduction band with one absorbed photon. In WTe_2_, the semi-metal characteristic makes it possess higher electron concentration in the conduction band. The higher concentration of electrons has stronger reflection on the excitation light as shown in Fig. 3. Therefore, compared to the binary component of WS_2_ and WTe_2,_ WS_1.76_Te_0.24_ has stronger photon absorption at 1.0 µm. The stronger photon absorption of WS_1.76_Te_0.24_ can increase the number of absorbed photons to produce more electrons at the same laser intensity. Eventually, the saturable intensity of WS_1.76_Te_0.24_ is lowered.

Suppose at a certain photon frequency, optical absorption satisfies $\frac{{\rm{d}}I}{{\rm{d}}z^{\prime} }=-\alpha (I)I$. Here the absorption coefficient *α(I)* is expressed as $\alpha (I)={\alpha }_{0}+{\alpha }_{NL}I$. *dz*′ is the propagation distance in the sample. The third-order nonlinear optics susceptibility Imχ^(3)^ can be expressed as

2 $${{\rm{Im}}{\rm{\chi }}}^{3}({\rm{esu}})=({10}^{-7}{{\rm{c}}{\rm{\lambda }}{\rm{n}}}^{2}/96{{\rm{\pi }}}^{2}){{\rm{\alpha }}}_{{\rm{NL}}}({\rm{cm}}/{\rm{W}})$$

here c is the speed of light, λ is the laser wavelength, n is the refractive index, the discrepancy caused by the linear absorption, namely figure of merit (FOM): ${\rm{FOM}}=|Im{\chi }^{(3)}/{\alpha }_{0}|$. Based on the model 2, we can obtain WS_1.76_Te_0.24_ with α_NL_ ~ −10^4^ cm/GW, Imχ^(3)^ ~10^−7^ esu, FOM ~10^−14^ cm• esu. Compared to previous works, the FOM of WS_1.76_Te_0.24_ perform one order of magnitude larger than that of grapheme, graphene oxide, MoS_2_/NMP dispersions ~10^−15^esu cm^16^. That suggests a promising potential to achieve efficient nonlinear performance by alloying TMDs. However, one should note that FOM varies with the different experiment conditions such as the wavelength, pulse width and so on. For convincing comparison, we carried out the Z-scan on the same condition and the nanosheets were prepared by the same parameters of liquid-phase exfoliation and spin-coating technique. The results are shown in Table 1. It is unambiguous that the FOM value of WS_1.76_Te_0.24_ SA was larger than those of WS_2_ and WTe_2_ SAs, which indicated the enhanced nonlinear performance of WS_1.76_Te_0.24._

The pump-probe system was adopted to study the carrier relaxation that reflects the optical response of materials. The undegenerated pump probe system is easy to align and the relaxation time is corresponded to the carrier-phonon coupling^28^. The ultrafast signal was measured using a Ti: Sapphire laser with pulse duration of 120 fs, repetition rate of 76 MHz, fluence of 200 µJ/cm^2^ at 395 nm as the pump and the probe beam was at 790 nm with much lower fluence. The probe reflection was a function of the delay time that was detected by a Si photodetector and amplified by a lock-in amplifier. As shown in Fig. 2e, the ultrafast signal of WS_1.76_Te_0.24_ flakes with absorption bleaching was obtained. The signal amplitude was as large as ~ 250%, implying excellent nonlinear optics property. Notably, the decay time was 3.8 ps fitted by a single exponential function. The decay time of WS_1.76_Te_0.24_ was significantly shorter than 13 ps of WS_2_^29^ and 5 ps of WTe_2_^6^, as a result of higher density of trapping states induced by Te doping in the nanoflake^30^.

### Investigation of the WS_1.76_Te_0.24_ saturable absorption in passively Q switched laser at 1.0 μm

**Figure 4:**
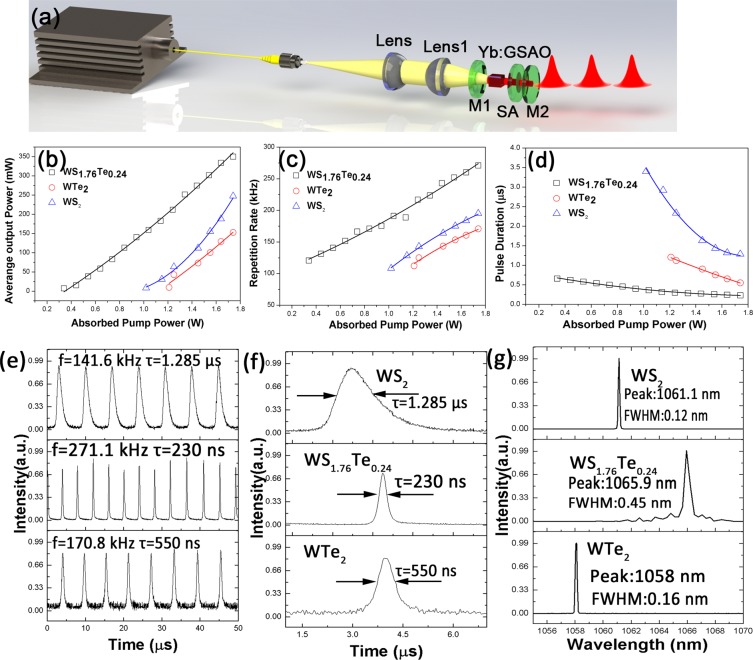
(**a**) Experimental setup of the LD pumped passively Q-switched Yb: GSAO laser at 1060 nm. (**b**)The output powers with increasing incident pump powers. (**c**) Pulse repetition rates. (**d**) Variations of pulse duration with increasing pump powers. The Q-switched lasers performance by the WS_2,_ WS_1.76_Te_0.24_ and WTe_2_ SAs: (**e**) the oscilloscope tracings. (**f**) the single pulse tracings (**g**) the optical spectra.

To identify whether the boosted saturable absorption effect of WS_1.76_Te_0.24_ did favor in Q-switched laser, we set up a passively Q-switched Yb: Gd_2_SrAl_2_O_7_ (Yb: GSAO) laser to investigate the performance of WS_2,_ WS_1.76_Te_0.24_ and WTe_2_ SAs. In Fig. 4a, the schematic of experiment setup was shown. A laser diode of 976 nm was served as pump source, which was coupled in a fiber of a core diameter of 105μm and the numerical aperture of 0.22. A doublet lens was employed to focus the beam at 105 μm within the Yb:GSAO crystal. In a cooled down system, the Yb: GSAO gain medium was wrapped with indium foil and mounted in a copper holder with water-cooled at 21 °C. The 11 mm linear cavity composed of 1060 nm high reflectivity M1(R = −200 mm) and 18% transmittance plano M2. The as-prepared three samples on SiO_2_ were inserted into the cavity serving as the saturable absorber.

Figure 4b–d show the characteristics of average output power, repetition rates and pulse durations of the lasers on absorbed pump power variation. The absorbed pump power thresholds of WS_2,_ WTe_2,_ and WS_1.76_Te_0.24_ were 1.02, 1.21 and 0.34 W_,_ respectively. The threshold of the WS_1.76_Te_0.24_ SA decreased to 28% due to the lower saturable intensity, as shown in Table 1. The output power was measured and calculated to be linearly correlated with the pump power. The maximum output powers of WS_2,_ WTe_2_ and WS_1.76_Te_0.24_were 247.5, 152.8 and 350 mW_,_ respectively. It is worth noting that WS_1.76_Te_0.24_ SA achieved twice larger output power of WTe_2_. The repetition rate continuously increased from 108.2 to 195.2 kHz for WS_2_ SA, and the repetition rate range of WS_1.76_Te_0.24_ SA was 120.8 to 271.1 kHz, scope of WTe_2_ varied from 112.2 to 170.8 kHz. As shown in Fig. 4d–f, pulse widths of 1.285 µs, 230 ns and 550 ns were obtained in 1.06 µm Q-switched lasers based on WS_2_, WS_1.76_Te_0.24_ and WTe_2_ SAs, respectively. As shown in Fig. 4e, the typical Q-switched pulse trains of WS_2,_ WS_1.76_Te_0.24_ and WTe_2_ were recorded by a 500 MHz bandwidth oscilloscope (Tektronix, DPO7054) through a high-speed detector (Thorlabs, DET10C/M), which confirms the stability of Q-switched operation. The narrowest pulse duration was obtained by WS_1.76_Te_0.24_ SA that should be attributed to the much larger modulation depth than WS_2_ and WTe_2_ (Fig. 2d and Table 1). The passively Q-switched laser based on WS_1.76_Te_0.24_ narrowed pulse duration to 18%.The optical spectra of the Q-switched lasers were measured with an infrared optical spectrum analyzer (Yokogawa, AQ-6315A) with a resolution of 0.05 nm. The wavelength of WS_2,_ WS_1.76_Te_0.24_ and WTe_2_ were centered at 1061.13 nm with 0.12 nm full width at half maximum (FWHM), 1065.93 nm with 0.45 nm FWHM, and 1058.01 nm with 0.16 nm FWHM, respectively. Q-switched laser based on the WS_1.76_Te_0.24_ SA can improve the key parameters as pulse width, slope efficiency and the average output power as listed in Table 2.

**Table 2:** Experiment results of Q-switched lasers based on three SAs.

Sample	WS_2_	WS_1.76_Te_0.24_	WTe_2_
Wavelength (nm)	1061.1	1065.9	1058.0
Output power (mW)	247.5	350	152.8
Pulse Duration	1.285 µs	230 ns	550 ns
Repetition rate (kHz)	141.6	271.1	170.8

## Conclusion

In this work, we have experimentally demonstrated the enhanced nonlinear optical properties of WS_1.76_Te_0.24_ by alloying WTe_2_ and WS_2_. We synthesized ternary WS_1.76_Te_0.24_ by CVT method. The SAED, EDS and Raman spectra showed good quality of the alloy WS_1.76_Te_0.24_ nanosheets. The saturable absorption of WS_1.76_Te_0.24_ at 1.06 µm was significantly more efficient than binary parents WTe_2_ and WS_2_ as evidenced by Z-scan and pump-probe results, where WS_1.76_Te_0.24_ SA showed 4 times deeper modulation depth, 28% lower saturable intensity and a much faster recovery time of 3.8 ps. The passively Q-switched laser based on WS_1.76_Te_0.24_ was found more efficient, with pulse duration narrowed to 18%, threshold decreased to 28% and output power enlarged twice. The doping engineering SAs can improve the Q-switched lasers performance with lower energy consumption, narrower pulse width, and larger average output power. The promising findings can provide a method to optimize performances of functional devices by doping engineering.

## Data Availability

Data Availability For original data, please contact xiezhenda@nju.edu.cn.
